# Phonomyography on Perioperative Neuromuscular Monitoring: An Overview

**DOI:** 10.3390/s22072448

**Published:** 2022-03-22

**Authors:** Yanjie Dong, Qian Li

**Affiliations:** Department of Anesthesiology, West China Hospital, Sichuan University, Chengdu 610041, China; 2020224025501@stu.scu.edu.cn

**Keywords:** phonomyography, acoustic myography, neuromuscular monitoring, neuromuscular blockade

## Abstract

Complications related to neuromuscular blockade (NMB) could occur during anesthesia induction, maintenance, and emergency. It is recommended that neuromuscular monitoring techniques be utilized perioperatively to avoid adverse outcomes. However, current neuromuscular monitoring methods possess different shortcomings. They are cumbersome to use, susceptible to disturbances, and have limited alternative monitoring sites. Phonomyography (PMG) monitoring based on the acoustic signals yielded by skeletal muscle contraction is emerging as an interesting and innovative method. This technique is characterized by its convenience, stable signal quality, and multimuscle recording ability and shows great potential in the application field. This review summarizes the progression of PMG on perioperative neuromuscular monitoring chronologically and presents the merits, demerits, and challenges of PMG-based equipment, aiming at underscoring the potential of PMG-based apparatuses for neuromuscular monitoring.

## 1. Introduction

Neuromuscular blockade (NMB), as the name implies, refers to a battery of agents that can specifically bind to the nicotinic receptor at the neuromuscular junction and thus block impulse transmission from the upper nerve to downstream muscle fibers, leading to transient or persistent skeletal muscle relaxation and aiding in easing endotracheal intubation and providing optimal conditions for operating and mechanical ventilation [1]. Many factors have been seen to affect the pharmacokinetics of NMB, such as age, organ function, or the apparent volume of distribution, requiring individualized NMB administration [2,3]. Hence, it is tricky for anesthesiologists to accurately maintain a shallow, moderate, or deep neuromuscular block at a proper time. However, an airway injury during endotracheal intubation, a sudden body-movement in surgical procedure, or a postoperative residual neuromuscular block (PRNB) during the reversal phase caused by the misjudgment of neuromuscular block may end in calamity [4,5,6]. Therefore, to reduce NMB-related complications, perioperative neuromuscular monitoring is highly recommended according to international guidelines to empower NMB with more controllability and to make NMB administration more individualized [7,8,9,10]. 

**Figure 1:**
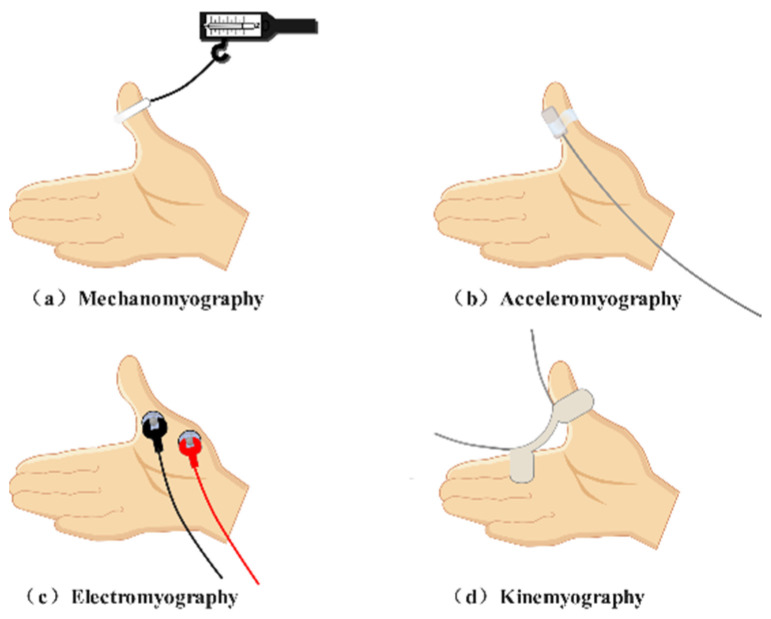
Typical neuromuscular monitoring patterns. (**a**) Mechanismography directly measures the force generated by the skeletal muscle. (**b**) Acceleromyography evaluates acceleration yielded by muscle contraction. (**c**) Electromyography records compound action potentials from the skeletal muscle. (**d**) Kinemyography employs a piezoelectric crystal to reflect muscle contraction.

Typical perioperative neuromuscular monitoring methods (Figure 1) include acceleromyography (ACC), mechanomyography (MMG), electromyography (EMG), and kinemyography (KMG) [11]. ACC-based equipment chooses a sensor that can evaluate acceleration yielded by muscle contraction [12]. ACC can be applied in many muscles, such as the adductor pollicis muscle, the orbicularis oculi muscle, and the corrugator supercilii muscle [13]. It is currently regarded as the gold standard for scientific research and clinical practice. MMG, which measures force generated by muscle contraction, was once considered the gold standard of neuromuscular monitoring; however, it has been phased out due to its bulky setup. EMG-based equipment records compound action potentials from the target muscle. This technique is a desirable choice for neuromuscular monitoring in clinical settings and is also able to detect neuromuscular blocks at various muscles. KMG-based facilities utilize one kind of piezoelectric mechanosensory to obtain and reflect the contraction of the adductor pollicis muscle [13]. This method is easy to use and clinically reliable.

However, frequently used neuromuscular monitoring patterns are always criticized for their diverse shortcomings (Table 1). Despite its universal utilization in clinical settings, ACC is susceptible to artifacts and will probably generate an overrated block degree [12,14]. A conventional ACC-based apparatus records the monaxial movement of the target muscle; therefore, signal quality relies heavily on the posture of the recording sites [15]. Recently, ACC-based equipment capable of estimating the multiplanar movement of the thumb has been developed. However, its feasibility exploration is still in progress [16]. The application of MMG demands a special posture of the hand and an elaborate setup [17]. In addition, this method can only record signals at the adductor pollicis muscle [18]. For EMG, the working principle limits its application intraoperatively owing to disturbances from other electronic equipment in the operating room [19]. In addition, the recording result of the EMG is an electromechanical compound instead of absolute mechanical contraction of skeletal muscle. Similar to MMG, KMG is also subjected to hand position [11]. Moreover, KMG is not interchangeable with MMG according to existing investigations [20].

Phonomyography (PMG), also named acoustic myography, is a little-known neuromuscular monitoring technique. However, previous studies suggested that PMG may be a feasible alternative neuromuscular monitoring method. Our paper provides an overview of the evolutionary trajectory of PMG on perioperative neuromuscular monitoring chronologically and summarizes the advantages and drawbacks of PMG as a means to present an avenue for further research. We performed a PubMed search using the keywords “phonomyography”, “acoustic myography”, and “muscle sounds” updated to December 2021. Articles describing PMG on perioperative neuromuscular monitoring are displayed in Table 2.

## 2. The Development of PMG-Based Equipment for Perioperative Neuromuscular Monitoring

**Figure 2:**
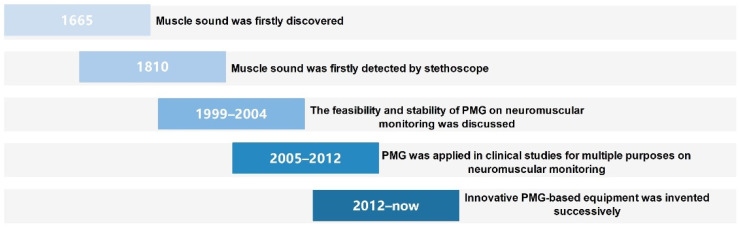
Timeline for the progression of PMG on perioperative neuromuscular monitoring.

Although PMG has been extensively discussed in the study of muscle contraction [36], myopathies [37], muscle fatigue [38], muscle pain [37] and prosthesis control [39,40], this technique is still poorly understood in the neuromuscular monitoring domain. Previous studies have already unveiled part of its properties when considering this technique as a method to detect the depth of neuromuscular block. The history of PMG-based equipment can be roughly divided into two categories (Figure 2): (1). Theoretical derivation and early exploration; (2). Applied research and technique renovation.

### 2.1. Theoretical Derivation and Early Exploration

#### 2.1.1. The Appearance of PMG

The voice emitted by the contraction of muscle fiber was described by Grimaldi primitively in 1665 [41]. By making a fist next to his ear, Grimaldi perceived this phenomenon and likened this kind of sound to thunderclap [42]. Then, it was not until 1810 that Wollaston confirmed the existence of that sound by a stethoscope [43]. During that period, people’s recognition of this kind of acoustic signal remained elusive and superficial.

In 1948, Gordon and Holbourn applied a crystal microphone and a piezoelectric microphone to obtain sound signals from human corrugator supercilii muscle [44]. They supposed that the acoustic waves emitted by skeletal muscle could reflect the activation of the motor unit. Later, Oster and Jaffe chose an air-coupled microphone and a contact microphone along with a transistorized stethoscope to detect these mysterious sounds in 1980. They gathered a series of sounds with a dominant frequency of 25 ± 5 Hz and drew the groundbreaking conclusion by underwater tests and insistent muscle contraction experiments. The sound signals created by the contraction of human skeletal muscle were the intrinsic property of the muscle itself rather than a byproduct of blood flow [42]. Their findings unmasked the essence of this phenomenon and shed light on further studies. Soon afterward, “acoustic myography” surfaced as a novel terminology. Five years after Oster and Jaffe’s experiment, another fundamental achievement was made. Barry’s team succeeded in testifying their hypothesis that PMG was purely the reflection of mechanical activity of muscle contraction rather than an electromechanical compound [45]. PMG was then discussed extensively during this period on the topic of muscle fatigue, normal muscle function evaluation, and prosthesis [38,40,46,47,48,49,50,51,52,53,54]. Other recording devices for PMG, such as phonocardiography, were also employed [45]. Nevertheless, achievement transformation was not realized during this period.

#### 2.1.2. Conceptions Related to PMG on Perioperative Neuromuscular Monitoring

Due to different patterns of motor unit recruitment, muscle sounds created by voluntary muscle contraction and evoked muscle contraction are distinct, which brings about two extremes in which the clinical application of PMG may originate. Transcutaneous neuromuscular electrical stimulation, in other words, evoked muscle contraction, is required to achieve perioperative neuromuscular monitoring. Both experiments in animals and human bodies illustrated that the electrical stimulation of motor nerves generates sound signals from innervated muscles [55,56,57]. There exists such a hypothesis that pressure waves within the skeletal muscle may be triggered by the simultaneous contraction of a single muscle fiber and its subsequent deformation [55]. With those pressure waves between muscle fibers integrated naturally, muscle sounds with polyphasic waveforms emerged. As presented above, PMG was established in such a theory: muscle contraction by lateral movement of muscle fibers can create acoustic signals with a low frequency, and the signals that occur can be transmitted to the surface of the skin. After collection and filtration, these sound signals will be transferred into electric signals, which can be evaluated quantitatively [12,45]. Interestingly, in almost all biomedical engineering-related domains, the term PMG is regarded as part of MMG. Some scholars believe MMG refers to the essence of mechanical contraction of skeletal muscle, while PMG, along with ACC and KMG, is the same technology with different sensors [58]. This point of view emphasizes the intrinsic characteristic of muscle sounds. Nevertheless, PMG stands alongside MMG, ACC, EMG and KMG in the neuromuscular monitoring domain [59]. The definition of these terminologies reflects the dissimilarity of muscle strength recorded by different kinds of sensors and is doubtless closer to the clinical practice of perioperative medicine.

Actually, the waveform of PMG obtained in evoked human skeletal muscle is biphasic with a 5–15 ms duration [44]. By analyzing the time and frequency domains of the PMG, several parameters, such as the root mean square, peak-to-peak amplitude, and frequency variance could be obtained [60]. Early investigations found that the root mean square amplitude of muscle sound is proportionally associated with the force of muscle contraction [61]. This simple and useful index was then utilized when PMG was introduced into the neuromuscular monitoring domain.

There are a few points that need to be noted when applying PMG to perioperative neuromuscular monitoring. First, unlike voluntary muscle contraction, the time interval between electric stimulation and acoustic signals is a special phenomenon in evoked muscle contraction. Studies have discovered that the time intervals are repeatable but not consistent in different muscles. For instance, the sound signal was 3.6 ± 1.0 ms after electric stimulation when the median nerve was stimulated, and the sound signal was 3.9 ± 1.1 ms after electric stimulation when the ulnar nerve was stimulated [56]. Although variation exists in time intervals, such distinctness in milliseconds is clinically acceptable. In addition, the amplitude of muscle sound at the muscle belly and tendon of the same muscle is different [51]. In addition, the thickness of subcutaneous fat may affect the quality of the transmission of muscle sounds [62].

In other words, since the intensity of electrical stimulation applied to the nerve can influence the number of motor units recruited, maximal stimulation is able to activate all motor units [63]. In other words, supramaximal nerve stimulation at a superficial muscle by a special stimulation mode may trigger repeatable and stable acoustic signals that would be feasible to evaluate neuromuscular blocks.

#### 2.1.3. Feasibility Exploration of PMG-Based Equipment

A feasible neuromuscular monitoring method is supposed to precisely reflect the pharmacokinetics of the administered NMB, both from onset to maximal effect and from maximal effect to offset. The initial exploration of PMG utilization in the neuromuscular monitoring domain did not generate satisfactory results. This may be attributed to defects in the microphones. After technical improvement of the sound detectors, the reliability and validity of PMG-based equipment turned out to be acceptable.

In 1999, Dascalu and his colleagues conducted the first experiment aiming at bearing out the clinical feasibility of PMG on perioperative neuromuscular monitoring. They compared PMG with MMG, EMG, and ACC among 25 anesthetized patients undergoing abdominal or orthopedic surgery [21]. Instead of using contact microphones, they utilized an air-coupled microphone to obtain sound signals. This sound detector, together with other equipment on the same hand as one subject, was intended to assess the depth of neuromuscular blockade at the adductor pollicis muscle. The data were seemingly uplifting, as the correlation coefficients of the T1/Tc ratio (stimulated by TOF after and before administration of nondepolarizing NMB) between the PMG and each other method were 0.862 (compared with MMG), 0.847 (compared with EMG), and 0.906 (compared with ACC). Indeed, this investigation possessed a range of shortcomings as they administered tubocurarine, atracurium, or succinylcholine to different patients, yet it did make a great start for PMG’s subsequent trekking to the neuromuscular monitoring sphere. Just one year later, Bellemare and his colleagues published another article focused on comparing the effect of PMG and MMG on the depth of neuromuscular block at the adductor pollicis muscle in 13 anesthetized subjects [22]. Unfortunately, PMG displayed a different pharmacodynamic property of the experimental muscle relaxant compared to MMG. Although the time to maximal effect of the administered NMB showed little difference between the two devices, PMG displayed slower onset and a faster recovery, which means PMG may overestimate neuromuscular block during anesthesia emergency, making the patients exposed to a risk of PRNB.

Prior experiments seemed to overshadow PMG’s clinical performance. However, Hemmerling and his colleagues insisted on evidence of the hypothesis of PMG on neuromuscular monitoring via a set of comparative studies. We regard Dr. Hemmerling, with his unique insights, as the pioneer of this issue under discussion. Briefly speaking, they published two pivotal articles in 2004 sequentially. One compared PMG with MMG at the adductor pollicis muscle, the first dorsal interosseus muscle, and the hypothenar muscles on neuromuscular monitoring [27], while another only compared these two methods at the adductor pollicis muscle [28]. As a matter of fact, Hemmerling drew a totally paradoxical conclusion to prior studies. Both investigations obtained a slight bias and a good agreement of the onset, maximal effect, and offset of the administered NMB and, thus, showed that the PMG was equipped with a similar ability to monitor neuromuscular block at the adductor pollicis muscle as the MMG. Trager reconfirmed this result by comparing PMG with MMG and KMG on neuromuscular monitoring at the same muscle in 2006 (they also proved that PMG was interchangeable with KMG) [18]. This inconsistency could be explained by the fact that Hemmerling applied a different sound detector compared to his predecessors. Specifically, Dascalu and Bellemare made a microphone with an air chamber as their sound detection device, whereas Hemmerling put an eye on a small condenser microphone featured by its flat and direct adhesion to the target muscle. It is believed that the stable contact between the skin surface and the sound detector contributes greatly to the quality of the signal collected [64].

#### 2.1.4. Stability Exploration of PMG-Based Equipment

Although whether the onset time of the adductor pollicis muscle is slower than that of the diaphragm is still in dispute, the recovery time of the adductor pollicis muscle is indeed slower than the diaphragm when NMB was administered [15,65]. Therefore, this muscle gained enough attention from scholars when considering new techniques for neuromuscular monitoring [66,67]. Nevertheless, other muscles, such as the corrugator supercilii muscle, and the laryngeal muscle are frequently monitored in experimental and clinical practice [68,69,70]. To ensure the stability of PMG-based equipment and broaden the scope of its utilization, Hemmerling’s team thus paid attention to the aforementioned skeletal muscles. First, they applied PMG and ACC at the corrugator supercilii muscle to 20 patients under general anesthesia. In this study, they compared the pharmacodynamic data of mivacurium recorded by PMG-based and ACC-based equipment. However, the results implied that PMG was not interchangeable with ACC for measuring neuromuscular blockade at the corrugator supercilii muscle as PMG recorded a longer onset, a more pronounced maximal effect, and a faster recovery [23]. Second, given that the position of the microphone may influence the quality of sound signals, they processed another investigation to determine the best recording site for PMG at the corrugator supercilii muscle and concluded that the best recording site at this muscle for PMG is located between the anterior midline and the lateral part of the forehead, over the eyebrow. Besides, the result was in accordance with the previous one despite the acoustic sensor being attached to the best recording site [24]. Owing to the dissatisfactory extent of motion of the corrugator supercilii muscle, ACC monitoring may not be valuable and referential at this muscle. It must be realized that since the correctness of PMG monitoring at the adductor pollicis muscle had been evidenced, a preferable study design should be comparing the data recorded by PMG at the adductor pollicis muscle and the corrugator supercilii muscle. Third, in comparison with the cuff pressure method (an accurate method to measure laryngeal adductor muscles for neuromuscular blockade), Hemmerling inserted a small condenser microphone into the left vestibular folds to evaluate the blockade level of the laryngeal adductor muscles intraoperatively. PMG illustrated a promising capability to measure neuromuscular blockade at the laryngeal adductor muscles, which was consequently equal to the cuff pressure method [25].

### 2.2. Applied Research and Technique Renovation

After the feasibility and stability of PMG had been preliminarily confirmed by years of efforts, further applied research of this technique called for concrete practice. During this period, PMG serves as the only neuromuscular monitoring method in these studies.

#### 2.2.1. Applied Research of PMG-Based Equipment

In 2004, Hemmerling and his colleagues carried out clinical research aiming at exploring the characteristics of the laryngeal muscles using PMG by a condenser microphone. This study revealed that the acoustic signal generated by muscles located around the laryngeal area is not interchangeable and the vocal cords would regain their ability to close earlier than open as the NMB fades [29]. This illustrates that it is impossible to employ alternative choices for PMG monitoring of the laryngeal adductor muscle. Moreover, they discussed the staircase phenomenon at the corrugator supercilia muscle and the hand muscles according to data recorded through PMG-based equipment. It turned out that, when PMG recording was utilized, the staircase effect remained apparent at the hand muscles, such as the adductor pollicis muscle while the corrugator supercilii muscle did not present the above phenomenon [30]. In other words, the corrugator supercilii muscle is probably a better target muscle for neuromuscular monitoring as a shorter duration of staircase phenomenon signifies a more efficient calibration process. In 2008, Hemmerling published a paper that measured the change in the pharmacodynamics of mivacurium caused by its interaction with propofol by PMG [34]. A five-minute or a twenty-minute intravenous propofol infusion was followed by a single bolus of mivacurium. Moreover, PMG was sensitive enough to detect the augmentation of the potency of the administered NMB.

In addition, Hemmerling’s team made a great contribution to further explore the property of PMG-based equipment. It came out that PMG could be used for neuromuscular monitoring at the adductor pollicis muscles whether it is the dominant hand or not [33]. Investigations conducted on the human body suggested that electrically stimulated sound signals are different in diverse muscles [71]. Given that the constituent ratio of type I and type II fibers is different among skeletal muscles [72], finding an ideal target muscle for PMG recording becomes important. Hemmerling’s team demonstrated that the vastus medialis muscle was also a desirable site for PMG recording by a piezoelectric microphone when some special operations hindered our access to the adductor pollicis muscle or the corrugator supercilii muscle, such as hand operations and neurosurgical operations [32].

#### 2.2.2. Technique Renovation of PMG-Based Equipment

In retrospect of the history of PMG on perioperative neuromuscular monitoring, it was the renovation of equipment that has always paved the way for this monitoring method. We believe that the more extensive acceptance and utilization of PMG are destined to be accomplished by the more sophisticated monitoring facilities with a superior hardware and software system.

Wehbe and colleagues described a novel prototype named “Relaxofon” in 2012 [35]. “Relaxofon” was the first machine tailored for the measurement of muscle sounds perioperatively. The precision of “Relaxofon” was tested preliminarily according to the correlation of the TOF ratio machine-generated between two piezo-electric microphones placed at the adductor pollicis muscle and the corrugator supercilii muscle on one subject under general anesthesia. It seemed that “Relaxofon” holds the potential to be a clinical routine. Yet, further studies of this device are anticipated to substantiate its repeatability and stability. Moreover, they gave a description of their blueprint for a closed-loop intelligent drug delivery robot for general anesthesia in 2014 [73]. This system encompasses a neuromuscular monitoring module based on PMG. The above notwithstanding, “Relaxofon” did not succeed in breaking the clinician’s stereotype. It is unwieldy and lacks alternative recording sites though previous studies had opened up a battery of options.

Another innovative PMG equipment is “CURO” (www.myodynamik.com). Harrison tried to introduce this microphone into the assessment of muscle activity [74]. In addition, a study in 2019 [75] focused on muscular function measurement of the cerebral palsy population by “CURO” inspired us for its prospective adhibition for muscular monitoring purposes. In fact, “CURO” was invented to serve canines and equines and is now accessible commercially [76]. It takes advantage of other microphones in service due to the acoustic gel covering its contact surface and its wireless feature. This superiority makes it more sensitive and convenient for acoustic signals. Most interestingly, “CURO” is now able to display three parameters (E-score, S-score, and T-score) that are closely related to muscle contraction [77]. This is also known as the ESTi^®^ Score [75]. However, “CURO” can only evaluate voluntary muscle contraction rather than electrically stimulated muscle contraction. The appearance of ESTi^®^ Score offered a solution for feature extraction and information interpretation of acoustic signals.

## 3. Properties of PMG-Based Equipment for Neuromuscular Monitoring

### 3.1. Intrinsic Merits of PMG-Based Equipment

**Figure 3:**
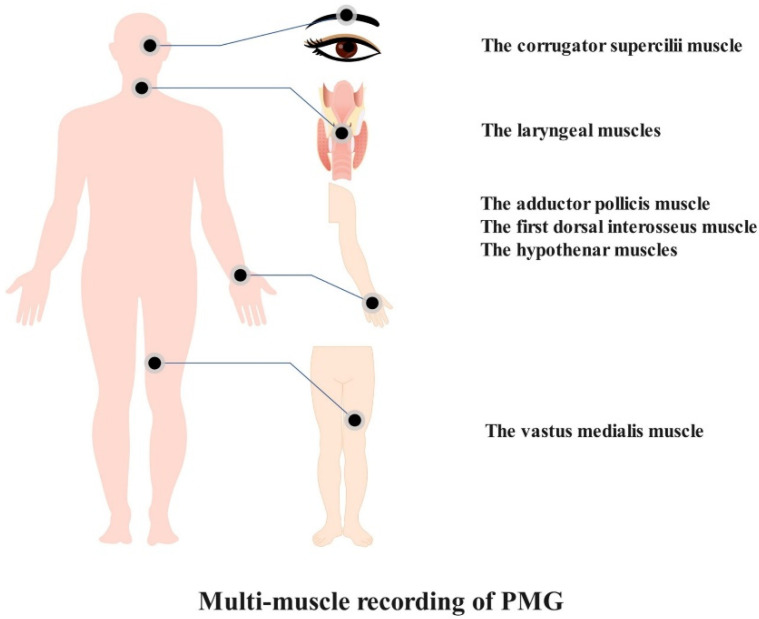
Alternative muscles for PMG-based neuromuscular monitoring.

The characteristics of PMG have embodied its advantages. The preponderance of PMG lies with its features of convenience and multimuscle recording (Figure 3). We speculate that PMG may possess some merits that would compensate for defects that belong to those extant neuromuscular monitoring methods and that may bridge the current technology gap. However, it remains to be determined whether PMG is truly a superior method due to the lack of related study. The properties of PMG in perioperative neuromuscular monitoring are as follows: (1) PMG-based equipment is easy to install and apply; (2) PMG-based equipment may present a stable signal that is barely affected by outside factors owing to its low frequency quality; and (3) PMG-based equipment is able to assess neuromuscular monitoring at the adductor pollicis muscle, corrugator supercilii muscle, vastus medialis muscle, first dorsal interosseus muscle, hypothenar muscles, and laryngeal muscles.

If neuromuscular monitoring machines keep on heading to a more complicated form, the popularization of perioperative neuromuscular monitoring would remain stagnant. We believe that the appearance of simple and user-friendly neuromuscular monitoring equipment will be achieved on account of PMG utilization.

### 3.2. Drawbacks and Challenges of PMG-Based Equipment

The shortboard of the PMG is equally obvious. On the one hand, the same target muscle of different individuals may create dissimilar muscle sounds. This can be explained by different muscle qualities, internal environments of muscle cells, and firing rates of motor units in different patients [78]. On the other hand, although high-frequency noises have been filtered, some coexisting low-frequency noises, such as heart sounds, breath sounds, and vascular sounds, may still be disturbances [79]. 

In addition, extant studies only paid attention to the comparison between PMG-based equipment and traditional neuromuscular monitoring methods. More in-depth explorations of PMG’s property on perioperative neuromuscular monitoring are required. Studies associated with different neuromuscular block degrees, anesthetic stages, and clinical populations are worth pursuing. In the first place, the benefits of deep neuromuscular block in laparoscopic abdominal surgery, cerebral aneurysm surgery, and laryngeal microsurgery have been exhibited [80,81,82]. Consequently, studies concerning the feasibility of PMG recording during deep neuromuscular block may not be avoided. In the second place, studies focused on the effect of PMG-based devices during rapid sequence induction or during anesthesia emergency with neuromuscular reversal agents are also needed. Last but not least, the consistency of PMG recording among different study populations, such as pediatric patients, young adults, and older patients and among different sub-population, such as obese patients and non-obese patients is required to be identified.

As the form of PMG-based equipment is still in the phase of the assembled prototype, problems related to signal gathering, analysis and display remain to be solved [20,41]. First, the improvement of sound detectors is the main focus. Recently, microphones with cylindrical and conical acoustic chambers have been developed [83]. This implies that air-coupled microphones will soon return to the stage center. In addition, the most advanced microphones in previous studies are piezoelectric microphones and condenser microphones with a diameter of 1.6 cm [18,25]. There is no question that current technology would give birth to more sophisticated sound detectors that are more suitable for gathering sound signals from other small muscles. It is worth noting that some delicate microphones with air chambers have been utilized in the study of prosthesis control [84]. Second, the data presented in previous studies are just filtered raw data. The method of data analysis is an unavoidable topic during the process of productization of PMG-based equipment. Information mining beyond amplitude from both the time and frequency domains of PMG is still a tricky problem. We believe artificial intelligence will be a potential choice. Third, as calibration endows ACC-based equipment with an individualized monitoring experience, PMG-based equipment may also need a calibration technique because of different muscle qualities and muscle masses in different individuals. Finally, only hand muscles, the corrugator supercilii muscle, the vastus medialis muscle, and the laryngeal muscles are not sufficient for the increasing number of surgical approaches. It is urgent to determine more alternative recording sites, such as the trapezius muscle, when the prone position is performed during nephrectomy or spinal surgery [85].

## 4. Discussion

Compared with traditional neuromuscular monitoring methods, PMG is considered an easy-to-use method and is believed applicable in multiple skeletal muscles. Another characteristic of PMG is the seemingly robust anti-interference ability because of its low-frequency nature. Recently, both advances in hardware systems and the software system of PMG puts this technology onto the stage again.

By comparison with frequently applied neuromuscular monitoring methods, the feasibility and stability of PMG on neuromuscular monitoring had been evidenced according to previous studies. Firstly, after a more reliable acoustic sensor without an air chamber was enrolled, the feasibility of PMG recording at the adductor pollicis muscle was shown to be interchangeable with MMG and KMG. Secondly, even though the best recording site was discovered, PMG could not display a similar degree of neuromuscular block as ACC at the corrugator supercilii muscle. However, the corrugator supercilii muscle may be a preferable target muscle for PMG owing to the unapparent staircase phenomenon. Thirdly, PMG recording is able to reflect neuromuscular block at the laryngeal adductor muscles by comparison with the cuff pressure method. Lastly, PMG monitoring at the vastus medialis muscle was also proved feasible. The above potentials of PMG-based equipment are deserving of further investigation and development.

In terms of present results, PMG is suitable for perioperative neuromuscular monitoring. Yet whether PMG is a superior method for neuromuscular monitoring remains to be proved. Drawbacks of PMG, such as inconsistency of acoustic signal among different individuals and interruptions of coexisting low-frequency noise within same individuals also require attention. Moreover, the aforementioned merits of PMG are still ambiguous due to limited studies, the lack of large-scale data, and the unstandardized study protocols. Besides, the modified Bland–Altman analysis which is designed for repeated measurements within the same individual was certainly not enrolled in previous studies. As the Good Clinical Practice guideline for NMB has been published, studies in the future should abide by the standard practice procedure to take peripheral or central body temperature and a recommended time point that may better represent the pharmacokinetics of NMB. The choice of the right statistic methods, such as a modified Bland–Altman analysis instead of a naïve version is also non-negligible. The evaluation of PMG-based devices should be extended to the exploration of their application in different neuromuscular degrees, anesthesia techniques, or clinical populations. In addition, we believe that PMG will gain a place in rehabilitative treatment and sports medicine soon or later, especially in the diagnosis, classification, and prognosis prediction for some neuromuscular diseases, such as sarcopenia. In a nutshell, PMG is a promising method for perioperative neuromuscular monitoring, but its journey to becoming a part of the clinical routine remains long and winding. 

## Figures and Tables

**Table 1:** Features of classical neuromuscular monitoring patterns.

Neuromuscular Monitoring Methods	Objects Detected	Recording Sites	Drawbacks
Acceleromyography	Acceleration	Adductor pollicis muscle Corrugator supercilii muscle …	Miscalculation of the block degree Susceptible to outside interference Elaborate setup
Mechanomyography	Force	Adductor pollicis muscle	Harsh conditions for the hand posture Adductor pollicis muscle only
Electromyography	Compound action potentials	Adductor pollicis muscle Corrugator supercilii muscle Orbicularis oculi muscle Laryngeal muscle Diaphragm…	Susceptible to electrical interference Not purely reflection of mechanical contraction of muscles
Kinemyography	Movement	Adductor pollicis muscle	Harsh conditions for the hand posture Adductor pollicis muscle only Not interchangeable with MMG

**Table 2:** Articles describing PMG on perioperative neuromuscular monitoring.

Author/Year	Sample Size	Sound Detector	Control	Muscle	Muscle Relaxant	Main Conclusion
Dascalu 1999 [21]	25	Air-coupled microphone	MMG EMG ACC	Adductor pollicis muscle	Tubocurarine Atracurium Succinylcholine	PMG could be used for perioperative neuromuscular monitoring
Bellemare 2000 [22]	13	Condenser microphone	MMG	Adductor pollicis muscle	Rocuronium	PMG was not an alternative method for neuromuscular monitoring at the adductor pollicis muscle when compared with MMG
Hemmerling 2002 [23]	20	Condenser microphone	ACC	Corrugator supercilii muscle	Mivacurium	PMG was not an alternative method for neuromuscular monitoring at the corrugator supercilii muscle when compared with ACC
Hemmerling 2002 [24]	27	Condenser microphone	ACC	Corrugator supercilii muscle	Mivacurium	The best recording site at the corrugator supercilii muscle for PMG is located between the anterior midline and the lateral part of the forehead, over the eyebrow
Hemmerling 2003 [25]	28	Condenser microphone	Cuff pressure method	Laryngeal adductor muscles	Mivacurium	PMG was an alternative method for neuromuscular monitoring at the laryngeal adductor muscles when compared with the cuff pressure method
Hemmerling 2004 [26]	15	Condenser microphone	Balloon pressure MMG	Corrugator supercilii muscle	Mivacurium	PMG was an alternative method for neuromuscular monitoring at the corrugator supercilii muscle when compared with Balloon pressure MMG
Hemmerling 2004 [27]	12	Condenser microphone	MMG	Hand muscles	Rocuronium	PMG was an alternative method for neuromuscular monitoring at hand muscles muscle when compared with MMG
Hemmerling 2004 [28]	28	Condenser microphone	MMG	Adductor pollicis muscle	Mivacurium	PMG was an alternative method for neuromuscular monitoring at the adductor pollicis muscle when compared with MMG
Hemmerling 2004 [29]	12	Condenser microphone	-	Posterior cricoarytenoid muscle /Lateral cricoarytenoid muscle	Mivacurium	The acoustic signals created by the posterior cricoarytenoid muscle and the lateral cricoarytenoid muscle after the administration of muscle relaxants are different.
Deschamps 2005 [30]	10	Piezo-electric microphone	MMG	Corrugator supercilii muscle/The first dorsal interosseus muscle	-	An apparent staircase phenomenon was found at the first dorsal interosseus muscle and the adductor pollicis muscle while no obvious staircase phenomenon occured at the corrugator supercilii muscle.
Hemmerling 2005 [31]	12	Piezo-electric microphone	-	Lateral cricoarytenoid muscle /Strap muscles of the neck	Mivacurium	PMG signals recorded were different outside and inside of the trachea for recovery time.
Michaud 2005 [32]	15	Piezo-electric microphone	-	Vastus medialis muscle Adductor pollicis	Mivacurium	The vastus medialis muscle is an alternative recording site for PMG
Michaud 2005 [33]	14	Piezo-electric microphone	-	Adductor pollicis muscle	Mivacurium	Whether it is the dominant hand would not influence the rustles of PMG recording at the adductor pollicis muscle
Trager 2006 [18]	14	Piezo-electric microphone	MMG KMG	Adductor pollicis muscle	Mivacurium	PMG was an alternative method for neuromuscular monitoring at the adductor pollicis muscle when compared with MMG or KMG
Hemmerling 2008 [34]	28	Piezo-electric microphone	-	Adductor pollicis muscle	Mivacurium	The potency of mivacurium is greater after a 20 min infusion of propofol compared with a 5 min infusion of propofol
Wehbe 2012 [35]	1	Piezo-electric microphone	-	Adductor pollicis /Corrugator supercilii muscle	Not mentioned	“Relaxofon” may be a feasible neuromuscular monitoring device

## Data Availability

No new data were created or analyzed in this study. Data sharing is not applicable to this article.

## References

[1] Bowman W.C. (2006). Neuromuscular Block. Br. J. Pharmacol..

[2] Larijani G.E., Gratz I., Silverberg M., Jacobi A.G. (1991). Clinical Pharmacology of the Neuromuscular Blocking Agents. DICP.

[3] Shakhnovich V., Smith P.B., Guptill J.T., James L.P., Collier D.N., Wu H., Livingston C.E., Zhao J., Kearns G.L., Benjamin D.K. (2018). Obese Children Require Lower Doses of Pantoprazole Than Nonobese Peers to Achieve Equal Systemic Drug Exposures. J. Pediatr..

[4] Chau I., Horn K., Dullenkopf A. (2020). Neuromuscular monitoring during modified rapid sequence induction: A comparison of TOF-Cuff^®^ and TOF-Scan^®^. Australas. Emerg. Care.

[5] Zhang X.-F., Li D.-Y., Wu J.-X., Jiang Q.-L., Zhu H.-W., Xu M.-Y. (2018). Comparison of deep or moderate neuromuscular blockade for thoracoscopic lobectomy: A randomized controlled trial. BMC Anesthesiol..

[6] Hunter J. (2017). Reversal of residual neuromuscular block: Complications associated with perioperative management of muscle relaxation. Br. J. Anaesth..

[7] Batistaki C., Vagdatli K., Tsiotou A., Papaioannou A., Pandazi A., Matsota P. (2019). A multicenter survey on the use of neuromuscular blockade in Greece. Does the real-world clinical practice indicate the necessity of guidelines?. J. Anaesthesiol. Clin. Pharmacol..

[8] Klein A.A., Meek T., Allcock E., Cook T.M., Mincher N., Morris C., Nimmo A.F., Pandit J.J., Pawa A., Rodney G. (2021). Recommendations for Standards of Monitoring During Anaesthesia and Recovery 2021: Guideline from the Association of Anaesthetists. Anaesthesia.

[9] Checketts M.R., Alladi R., Ferguson K., Gemmell L., Handy J.M., Klein A., Love N.J., Misra U.K., Morris C., Nathanson M.H. (2015). Recommendations for standards of monitoring during anaesthesia and recovery 2015: Association of Anaesthetists of Great Britain and Ireland. Anaesthesia.

[10] Söderström C.M., Eskildsen K.Z., Gätke M.R., Staehr-Rye A.K. (2017). Objective Neuromuscular Monitoring of Neuromuscular Blockade in Denmark: An Online-Based Survey of Current Practice. Acta Anaesthesiol. Scand..

[11] Hemmerling T.M., Le N. (2007). Brief review: Neuromuscular monitoring: An update for the clinician. Can. J. Anaesth..

[12] Murphy G.S. (2018). Neuromuscular Monitoring in the Perioperative Period. Anesth. Analg..

[13] Naguib M., Brull S., Johnson K.B. (2017). Conceptual and technical insights into the basis of neuromuscular monitoring. Anesthesia.

[14] Duţu M., Ivaşcu R., Tudorache O., Morlova D., Stanca A., Negoiţă S., Corneci D. (2018). Neuromuscular monitoring: An update. Rom. J. Anaesth. Intensiv. Care.

[15] Dahaba A.A., Suljevic I., Xiao Z.Y., Wang K. (2018). Mindray 3-directional NMT Module (a new generation “Tri-axial” neuromuscular monitor) versus the Relaxometer mechanomyograph and versus the TOF-Watch SX acceleromyograph. Int. J. Clin. Monit. Comput..

[16] Colegrave N., Billard V., Motamed C., Bourgain J.L. (2016). Comparison of the Tof-Scan™ Acceleromyograph to Tof-Watch Sx™: Influence of Calibration. Anaesth. Crit. Care Pain Med..

[17] Bowdle A., Jelacic S. (2020). Progress towards a standard of quantitative twitch monitoring. Anesthesia.

[18] Trager G., Michaud G., Deschamps S., Hemmerling T.M. (2006). Comparison of phonomyography, kinemyography and mechanomyography for neuromuscular monitoring. Can. J. Anaesth..

[19] Lee W. (2021). The latest trend in neuromuscular monitoring: Return of the electromyography. Anesth. Pain Med..

[20] Fuchs-Buder T., Claudius C., Skovgaard L.T., Eriksson L.I., Mirakhur R.K., Viby-Mogensen J. (2007). Good clinical research practice in pharmacodynamic studies of neuromuscular blocking agents II: The Stockholm revision. Acta Anaesthesiol. Scand..

[21] Dascalu A., Geller E., Moalem Y., Manoah M., Enav S., Rudick Z. (1999). Acoustic monitoring of intraoperative neuromuscular block. Br. J. Anaesth..

[22] Bellemare F., Couture J., Donati F., Plaud B. (2000). Temporal Relation between Acoustic and Force Responses at the Adductor Pollicis during Nondepolarizing Neuromuscular Block. Anesthesiology.

[23] Hemmerling T.M., Donati F., Babin D., Beaulieu P. (2002). Duration of control stimulation does not affect onset and offset of neuromuscular blockade at the corrugator supercilii muscle measured with phonomyography or acceleromyography. Can. J. Anaesth..

[24] Hemmerling T., Donati F., Beaulieu P., Babin D. (2002). Phonomyography of the corrugator supercilii muscle: Signal characteristics, best recording site and comparison with acceleromyography. Br. J. Anaesth..

[25] Hemmerling T.M., Babin D., Donati F. (2003). Phonomyography as a Novel Method to Determine Neuromuscular Blockade at the Laryngeal Adductor Muscles: Comparison with the Cuff Pressure Method. Anesthesiology.

[26] Hemmerling T.M., Michaud G., Babin D., Trager G., Donati F. (2004). Comparison of phonomyography with balloon pressure mechanomyography to measure contractile force at the corrugator supercilii muscle. Can. J. Anaesth..

[27] Hemmerling T.M., Michaud G., Trager G., Deschamps S. (2004). Phonomyographic measurements of neuromuscular blockade are similar to mechanomyography for hand muscles. Can. J. Anaesth..

[28] Hemmerling T.M., Michaud G., Trager G., Deschamps S., Babin D., Donati F. (2004). Phonomyography and Mechanomyography Can Be Used Interchangeably to Measure Neuromuscular Block at the Adductor Pollicis Muscle. Anesth. Analg..

[29] Hemmerling T.M., Michaud G., Trager G., Donati F. (2004). Simultaneous Determination of Neuromuscular Blockade at the Adducting and Abducting Laryngeal Muscles Using Phonomyography. Anesth. Analg..

[30] Deschamps S., Trager G., Mathieu P.A., Hemmerling T.M. (2005). The staircase phenomenon at the corrugator supercilii muscle in comparison with the hand muscles. Br. J. Anaesth..

[31] Hemmerling T.M., Michaud G., Deschamps S., Trager G. (2005). An External Monitoring Site at the Neck Cannot Be Used to Measure Neuromuscular Blockade of the Larynx. Anesth. Analg..

[32] Michaud G., Trager G., Deschamps S., Hemmerling T.M. (2005). Monitoring neuromuscular blockade at the vastus medialis muscle using phonomyography. Can. J. Anaesth..

[33] Michaud G., Trager G., Deschamps S., Hemmerling T.M. (2005). Dominance of the Hand Does Not Change the Phonomyographic Measurement of Neuromuscular Block at the Adductor Pollicis Muscle. Anesth. Analg..

[34] Hemmerling T.M., Le N., Decarie P., Cousineau J., Bracco D. (2008). Total intravenous anesthesia with propofol augments the potency of mivacurium. Can. J. Anaesth..

[35] Wehbe M., Mathieu P.A., Hemmerling T.M. Relaxofon: A neuromuscular blockade monitor for patients under general anesthesia. Proceedings of the 2012 Annual International Conference of the IEEE Engineering in Medicine and Biology Society.

[36] Jaskólska A., Madeleine P., Jaskólski A., Kisiel-Sajewicz K., Arendt-Nielsen L. (2007). A Comparison between Mechanomyographic Condenser Microphone and Accelerometer Measurements During Submaximal Isometric, Concentric and Eccentric Contractions. J. Electromyogr. Kinesiol..

[37] Madeleine P., Arendt-Nielsen L. (2005). Experimental muscle pain increases mechanomyographic signal activity during sub-maximal isometric contractions. J. Electromyogr. Kinesiol..

[38] Barry D.T., Gordon K.E., Hinton G.G. (1990). Acoustic and surface EMG diagnosis of pediatric muscle disease. Muscle Nerve.

[39] Alves N., Chau T. (2010). Uncovering patterns of forearm muscle activity using multi-channel mechanomyography. J. Electromyogr. Kinesiol..

[40] Barry D.T., Leonard J.A., Gitter A.J., Ball R.D. (1986). Acoustic myography as a control signal for an externally powered prosthesis. Arch. Phys. Med. Rehabil..

[41] Martinez A.P., Moser T.P., Saran N., Paquet M., Hemmerling T., Berry G.K. (2017). Phonomyography as a non-invasive continuous monitoring technique for muscle ischemia in an experimental model of acute compartment syndrome. Injury.

[42] Oster G., Jaffe J. (1980). Low frequency sounds from sustained contraction of human skeletal muscle. Biophys. J..

[43] Hemmerling T.M., Babin D. (2002). Phonomyography--acoustic myography using condenser microphones: A promising new method of monitoring neuromuscular transmission. Anaesth. Intensiv. Care.

[44] Gordon G., Holbourn A.H.S. (1948). The sounds from single motor units in a contracting muscle. J. Physiol..

[45] Barry D.T., Geiringer S.R., Ball R.D. (1985). Acoustic myography: A noninvasive monitor of motor unit fatigue. Muscle Nerve.

[46] Stokes M., Dalton P.A. (1991). Acoustic myography for investigating human skeletal muscle fatigue. J. Appl. Physiol..

[47] Dalton P., Comerford M., Stokes M. (1992). Acoustic myography of the human quadriceps muscle during intermittent fatiguing activity. J. Neurol. Sci..

[48] Ouamer M., Boiteux M., Petitjean M., Travens L., Salès A. (1999). Acoustic myography during voluntary isometric contraction reveals non-propagative lateral vibration. J. Biomech..

[49] Dalton P.A., Stokes M.J. (1991). Acoustic myography reflects force changes during dynamic concentric and eccentric contractions of the human biceps brachii muscle. Eur. J. Appl. Physiol. Occup. Physiol..

[50] Goldenberg M.S., Yack H.J., Cerny F.J., Burton H.W. (1991). Acoustic myography as an indicator of force during sustained contractions of a small hand muscle. J. Appl. Physiol..

[51] Stokes M., Dalton P. (1991). Acoustic myographic activity increases linearly up to maximal voluntary isometric force in the human quadriceps muscle. J. Neurol. Sci..

[52] Lee D.J., Stokes M.J., Taylor R.J., Cooper R.G. (1992). Electro and acoustic myography for noninvasive assessment of lumbar paraspinal muscle function. Eur. J. Appl. Physiol. Occup. Physiol..

[53] Petitjean M., Ripart J., Couture J., Bellemare F. (1997). Diaphragmatic fatigue investigated by phonomyography. Am. J. Respir. Crit. Care Med..

[54] Petitjean M., Maton B., Cnockaert J.C. (1992). Evaluation of human dynamic contraction by phonomyography. J. Appl. Physiol..

[55] Brozovich F., Pollack G. (1983). Muscle contraction generates discrete sound bursts. Biophys. J..

[56] Barry D.T. (1991). Muscle sounds from evoked twitches in the hand. Arch. Phys. Med. Rehabil..

[57] Marchetti M., Felici F., Bernardi M., Minasi P., Di Filippo L. (1992). Can Evoked Phonomyography Be Used to Recognize Fast and Slow Muscle in Man?. Int. J. Sports Med..

[58] Orizio C., Gobbo M. (2006). Mechanomyography. Wiley Encyclopedia of Biomedical Engineering.

[59] Murphy G.S., Brull S.J. (2021). Quantitative Neuromuscular Monitoring and Postoperative Outcomes: A Narrative Review. Anesthesiology.

[60] Ibitoye M.O., Hamzaid N.A., Zuniga J.M., Hasnan N., Wahab A.K.A. (2014). Mechanomyographic Parameter Extraction Methods: An Appraisal for Clinical Applications. Sensors.

[61] Petitjean M., Bellemare F. (1994). Phonomyogram of the diaphragm during unilateral and bilateral phrenic nerve stimulation and changes with fatigue. Muscle Nerve.

[62] Esposito F., Veicsteinas A., Orizio C., Malgrati D. (1996). Time and frequency domain analysis of electromyogram and sound myogram in the elderly. Eur. J. Appl. Physiol. Occup. Physiol..

[63] Adams G.R., Harris R.T., Woodard D., Dudley G.A. (1993). Mapping of electrical muscle stimulation using MRI. J. Appl. Physiol..

[64] Bolton C.F., Parkes A., Thompson T.R., Clark M.R., Sterne C.J. (1989). Recording sound from human skeletal muscle: Technical and physiological aspects. Muscle Nerve.

[65] Moerer O., Bittner J., Hinz J., Sydow M. (2005). Effect of Rocuronium on the Diaphragm, Musculus Adductor Pollicis and Orbicularis Oculi in Two Groups of Different Age. Anasthesiol. Intensivmed. Notfallmed. Schmerzther..

[66] Krijtenburg P., Honing G., Martini C., Olofsen E., Van Elst H., Scheffer G., Dahan A., Keijzer C., Boon M. (2019). Comparison of the TOF-Cuff^®^ monitor with electromyography and acceleromyography during recovery from neuromuscular block. Br. J. Anaesth..

[67] Kameyama Y., Takagi S., Seto K., Kajiwara I., Goto M., Kitajima O., Suzuki T. (2018). Efficiency of the TOF-Cuff™ for the evaluation of rocuronium-induced neuromuscular block and its reversal with sugammadex: A comparative study vs. acceleromyography. J. Anesth..

[68] Hemmerling T.M., Donati F. (2003). Neuromuscular blockade at the larynx, the diaphragm and the corrugator supercilii muscle: A review. Can. J. Anaesth..

[69] Dieye E., Minville V., Asehnoune K., Conil C., Georges B., Cougot P., Fourcade O., Conil J.-M. (2014). Pharmacodynamics of cisatracurium in the intensive care unit: An observational study. Ann. Intensive Care.

[70] Viby-Mogensen J. (2001). Neuromuscular Monitoring. Curr. Opin. Anaesthesiol..

[71] Orizio C. (1993). Muscle sound: Bases for the introduction of a mechanomyographic signal in muscle studies. Crit. Rev. Biomed. Eng..

[72] Lexell J., Taylor C.C. (1991). Variability in muscle fibre areas in whole human quadriceps muscle: Effects of increasing age. J. Anat..

[73] Wehbe M., Arbeid E., Cyr S., Mathieu P.A., Taddei R., Morse J., Hemmerling T.M. (2014). A technical description of a novel pharmacological anesthesia robot. Int. J. Clin. Monit. Comput..

[74] Harrison A.P., Danneskiold-Samsøe B., Bartels E.M. (2013). Portable acoustic myography—A realistic noninvasive method for assessment of muscle activity and coordination in human subjects in most home and sports settings. Physiol. Rep..

[75] Pingel J., Andersen I.T., Broholm R., Harder A., Bartels E.M., Bülow J., Harrison A. (2019). An acoustic myography functional assessment of cerebral palsy subjects compared to healthy controls during physical exercise. J. Muscle Res. Cell Motil..

[76] Riis K., Harrison A., Riis-Olesen K. (2013). Non-invasive assessment of equine muscular function: A case study. Open Vet. J..

[77] Harrison A.P. (2018). A more precise, repeatable and diagnostic alternative to surface electromyography—An appraisal of the clinical utility of acoustic myography. Clin. Physiol. Funct. Imaging.

[78] Ibitoye M.O., Hamzaid N.A., Zuniga J.M., Wahab A.K.A. (2014). Mechanomyography and muscle function assessment: A review of current state and prospects. Clin. Biomech..

[79] Silva J., Chau T. (2005). A Mathematical Model for Source Separation of MMG Signals Recorded With a Coupled Microphone-Accelerometer Sensor Pair. IEEE Trans. Biomed. Eng..

[80] Murphy G.S. (2021). Pro: Deep Neuromuscular Blockade Should be Maintained During Laparoscopic Surgery. Anaesth. Crit. Care Pain Med..

[81] Kim B.Y., Chung S.H., Park S.-J., Han S.-H., Kwon O.-K., Chung J.-Y., Kim J.-H. (2020). Deep neuromuscular block improves angiographic image quality during endovascular coiling of unruptured cerebral aneurysm: A randomized clinical trial. J. Neurointerv. Surg..

[82] Kim H.J., Lee K.-Y., Park W.K., Lee B.R., Joo H.M., Koh Y.W., Seo Y.W., Kim W.S., Yoo Y.C. (2015). Deep neuromuscular block improves the surgical conditions for laryngeal microsurgery. Br. J. Anaesth..

[83] Posatskiy A., Chau T. (2012). Design and evaluation of a novel microphone-based mechanomyography sensor with cylindrical and conical acoustic chambers. Med. Eng. Phys..

[84] Posatskiy A., Chau T. (2012). The effects of motion artifact on mechanomyography: A comparative study of microphones and accelerometers. J. Electromyogr. Kinesiol..

[85] Oh S.K., Park S., Lim B.G., Kim Y.S., Kim H., Kong M.H. (2021). Comparison between the trapezius and adductor pollicis muscles as an acceleromyography monitoring site for moderate neuromuscular blockade during lumbar surgery. Sci. Rep..

